# Author reply to letter to the editor: Beyond the ruler: Measuring what truly matters in EUS-guided pancreatic cancer genomics

**DOI:** 10.1055/a-2677-0491

**Published:** 2025-08-14

**Authors:** Junya Sato, Hirotoshi Ishiwatari, Kazuma Ishikawa, Hiroki Sakamoto, Masahiro Yamamura, Takuya Doi, Kazunori Takada, Yoichi Yamamoto, Masao Yoshida, Sayo Ito, Noboru Kawata, Kenichiro Imai, Kinichi Hotta, Hiroyuki Ono

**Affiliations:** 1 38471 Endoscopy, Shizuoka Cancer Center, Nagaizumi, Japan; 2 Gastroenterology and Hepatology, St. Marianna University School of Medicine, Kawasaki, Japan; 3 Endoscopy, Shizuoka Cancer Center, Nagaizumi, Japan



We are grateful to the authors of the Letter to the Editor for their thoughtful and constructive comments regarding our recent article, “Benefits of macroscopic on-site evaluation in endoscopic ultrasound-guided tissue acquisition for comprehensive genomic profiling. ” We appreciate this opportunity to respond to the points raised, which we address below.

Our study primarily aimed to identify an optimal specimen collection method for FoundationOne CDx (F1CDx) using endoscopic ultrasound-guided tissue acquisition (EUS-TA), ensuring sufficient material for comprehensive genomic profiling.

## Response to Dr. Wu's first point

First, as Dr. Wu rightly pointed out, the detailed criteria for F1CDx evaluation, particularly regarding "Qualified" results, remain undisclosed by the testing provider. It is widely recognized that a "Qualified" result suggests reduced sensitivity for detecting genomic alterations and signatures, potentially leading to an underreported tumor mutation burden. Our unpublished data from the same dataset of the article showed a significantly lower success rate for microsatellite instability analysis in “Qualified” cases compared with “Passed” cases (11% vs. 98%, *P* < 0.001).

Furthermore, only 11 of 75 cases, whether categorized as “Passed” or “Qualified”, ultimately led to eligibility for genomic therapy. In all instances in which genomic therapy could not be administered, the reason was absence of an approved drug with proven efficacy in Japan. This strongly suggests that the extremely low detection rate of actionable mutations in pancreatic cancer is the key factor limiting the transition to genomic therapy. These findings underscore the critical importance of appropriate specimen collection via EUS-TA for F1CDx as an initial, pivotal step toward identifying the small subset of pancreatic cancer patients who may harbor actionable mutations.

## Response to Dr. Wu's second point

Second, we appreciate your comment regarding the relationship between prior chemotherapy and appropriate macroscopic visible core (MVC) length. We conducted an additional analysis using the same dataset. This analysis revealed that the optimal MVC length in patients without prior chemotherapy was 36 mm (area under the curve (AUC) 0.531, 95% confidence interval [CI] 0.278–0.784), whereas in patients who had received chemotherapy before EUS-TA, it was 51 mm (AUC 0.537, 95% CI 0.206–0.868). As Dr. Wu suggested, in patients who have undergone chemotherapy, there may be an increased likelihood of obtaining necrotic or fibrotic tissue during sampling, which could necessitate a lengthier MVC for successful F1CDx analysis. However, our cohort included only 13 patients who had received chemotherapy prior to EUS-TA. Therefore, we believe that further investigation with a larger number of cases is essential to definitively determine whether prior chemotherapy before EUS-TA significantly impacts the appropriate MVC length required for F1CDx.

**Publication note**

Letters to the editor do not necessarily represent the opinion of the editor or publisher. The editor and publisher reserve the right to not publish letters to the editor, or to publish them abbreviated or in extracts.

